# Pure Orbital Floor Blow-in Fracture

**Published:** 2013-03-20

**Authors:** Joshua B. Elston, Jessica A. Ching, Matthew E. Hiro, Wyatt G. Payne

**Affiliations:** Plastic Surgery Section, Bay Pines VA Healthcare System, Bay Pines, FL; and the Division of Plastic Surgery, University of South Florida College of Medicine, Tampa, FL

## DESCRIPTION

A 55-year-old white man presented with double vision 2 days after low-velocity blunt force–isolated facial trauma. He complained of persistent vertical and horizontal double vision, particularly with inferior and lateral gaze. He had minimal dentition, but he believed that his teeth aligned normally. A computed tomographic scan demonstrated a right orbital floor fracture.

On physical examination, the patient had a significant component of vertical dystopia on the right side, which was initially attributed to his edema. He was observed for a week to see if his symptoms resolved. At follow-up, his vertical dystopia was still present despite resolution of periorbital edema. Upon surgical exploration, he was found to have an upwardly displaced orbital floor fragment that had trapped a portion of maxillary sinus mucosa.

At orbital exploration, the findings mentioned earlier were encountered. The maxillary sinus mucosa was reduced into its original position. The orbital floor fracture piece was easily reduced into anatomic position. A titanium plate was contoured to rigidly fixate the fracture fragments and was secured into place at the orbital rim. The globe was allowed to return to its anatomic position. It was noted that the patient's vertical dystopia was still present and that the plate was now the cause. The plate was removed and the patient was reexamined. His vertical dystopia appeared to have been corrected and the fracture fragment appeared stable. Postoperatively, our patient's diplopia resolved and he had minimally evident vertical dystopia at 3 months follow-up.

## QUESTIONS

1. **What classifies an orbital fracture as “pure” or “impure”?**
2. **What physical examination findings could suggest a blow-in type fracture?**
3. **What percentage of patients with blow-in fractures experience persistent diplopia?**
4. **How is this fracture similar and different to a pediatric “trap-door” fracture?**

## DISCUSSION

Orbital floor fractures can be described as “pure” or “impure” on the basis of the coexistent presence/absence of an orbital rim fracture. When the rim fractures, it often provides some decompression of the orbit by rotational displacement. When the rim remains fixed in the absence of fracture, the inwardly displaced fracture fragment acts as a space-occupying lesion and decreases intraorbital volume.^1^ Our patient had an intact orbital rim with upward displacement of his orbital floor that was held in place by a portion of maxillary sinus mucosa in a trap-door fashion that prevented self-reduction by gravity or after the edema had resolved. This resulted in a significant vertical dystopia, which caused persistent diplopia. It is important to recognize the telltale signs of a blow-in fracture. The classic signs of an orbital floor fracture are present: periorbital edema and ecchymoses, conjunctival injection, and hypesthesia in the maxillary distribution of the trigeminal nerve. However, with a blow-in fracture, superior displacement of the globe and proptosis on the fracture side are possibly present.^1^ These signs indicate a decrease in orbital volume. These findings coupled with CT imagery showing upwardly displaced fracture fragments make this diagnosis high in the differential and would necessitate urgent ophthalmologic evaluation and surgical reduction/decompression. The largest study to date of orbital blow-in fractures reported preoperative symptoms and postoperative complication rates in their 42 patients. Although nearly one third of patients presented with double vision, only 8% had persistent diplopia.^1^ A pure orbital floor blow-in fracture is somewhat analogous to the trapdoor fracture described in the pediatric population. This classically described pediatric fracture is caused by the relative elasticity of the pediatric skeleton that displaces the weaker middle floor downward, while the stronger medial or lateral orbital floor acts as a hinge. The inferior rectus muscle can become trapped in this fracture and requires urgent surgical exploration to prevent incarceration. The etiology of damage in both scenarios is caused by compression and ischemia of orbital tissue; however, it is important to understand the difference in presentation. Pediatric trap-door fractures will result in gaze restriction with normal to increased orbital volume leading to ischemia of an entrapped muscle. Orbital blow-in fractures will usually not have gaze restriction, but proptosis is the telltale sign indicating a decrease in orbital volume that causes diffuse compression of orbital contents and ischemia.

Orbital “blow-in” fractures were first described in 1964 by Dingman and Natvig after encountering a patient who received a severe blow to the maxillary sinus. X-ray film showed upward displacement of the orbital floor into the orbit.^2^ This type of fracture is much less common than the often encountered orbital floor “blow-out” fracture.^1^ The presentation in the few reports in the literature of patients with orbital floor blow-in fractures typically have malar trauma that results in a depression of the maxillary sinus.^1^^-^3 This can result in an upward displacement of orbital floor contents, which causes superior displacement of the globe. This presentation can be even more concerning than a blow-out fracture because it causes a decrease in orbital volume. This can lead to compression of orbital contents and subsequent ischemia or globe rupture from fracture fragments. This type of clinical picture is surgically managed in the same fashion as any other orbital floor fracture: adequate exposure, reduction of the fracture, restoring preinjury orbital volume, and determining the need for fixation of the fracture. In the initial report of a blow-in fracture described earlier^2^ the patient underwent exploration and the fracture was reduced without any fixation. As with any orbital floor fracture, if the fragments are unstable, rigid fixation will be required. However, if the fracture is well defined and is stable upon reduction, no fixation is necessary. When a patient presents with classic signs of orbital floor fracture including diplopia, periorbital edema, a palpable bony step off, and hypesthesia of the ipsilateral midface, a CT scan should be performed to evaluate the extent of the fracture. Incarcerated extraocular muscles should be suspected any time CT scan demonstrates the ligamentous sling below the globe is disrupted leading to flattening of the normally rounded inferior rectus muscle. These patients will have gaze restrictions and will possibly be enophthalmic on examination. Proptosis and a superior globe displacement resulting in dystopia on the injured side should point to the blow-in fracture and emergent ophthalmologic examination for possible vision salvaging reduction.

## Figures and Tables

**Figure 1:**
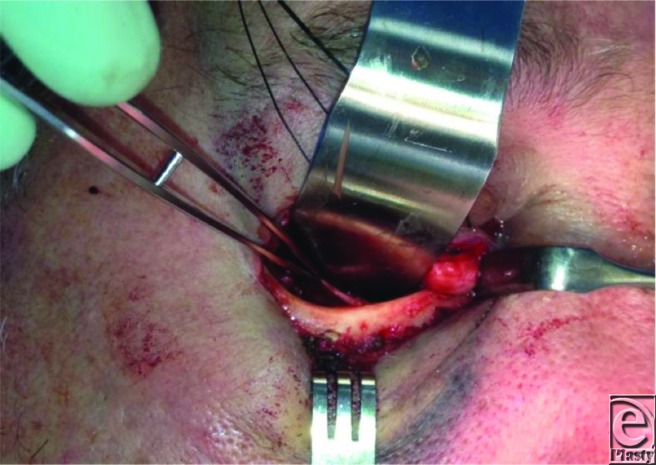
Intraoperative photo showing the displaced fracture fragment.

**Figure 2:**
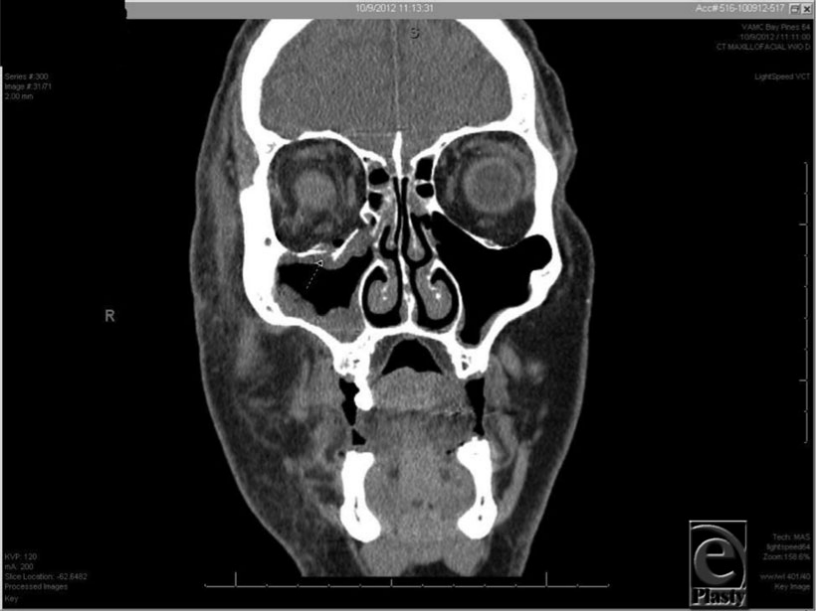
Coronal slicing demonstrates orbital floor fracture and herniation of maxillary sinus soft tissue into the orbit.

**Figure 3:**
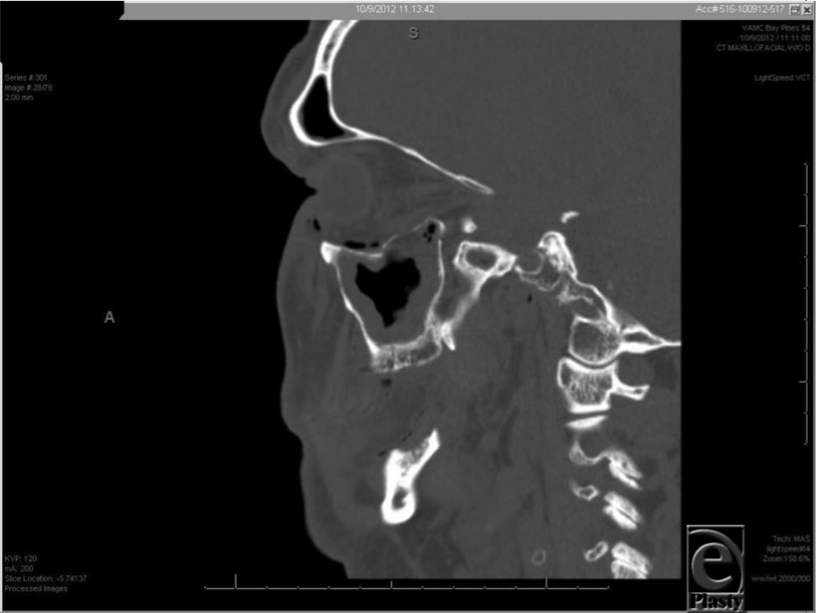
Orbital floor fracture and periorbital emphysema is visualized on sagittal views.
